# Parental mental health and risk of poor mental health and death by suicide in offspring: a population-wide data-linkage study

**DOI:** 10.1017/S2045796022000063

**Published:** 2022-04-19

**Authors:** A. Maguire, E. Ross, D. O'Reilly

**Affiliations:** Centre for Public Health, Queen's University Belfast, Belfast, BT12 6BJ, Northern Ireland

**Keywords:** Adolescence, epidemiology, poor mental health, record-linkage study, suicide

## Abstract

**Aims:**

Suicide is a major public health concern. Identifying those most at risk is vital to ensure the implementation of effective interventions. Mental health (MH) is known to have a genetic component and parental MH is associated with offspring MH. However, little is known about the effect of parental psychopathology on offspring suicide risk. The aim of this study is to determine if children living with parents with poor MH are at an increased risk of poor MH, or death by suicide.

**Methods:**

This population-wide cohort study linked data from the 2011 Northern Ireland Census to 6 years' death records (2011–2016). Information on MH status, in addition to other individual and household-level attributes, was derived from the 2011 Census. Logistic regression was utilised to examine the association between parental poor MH and offspring MH and suicide risk, with adjustment for socio-demographic characteristics.

**Results:**

Overall, 11.6% of the cohort of 618 970 individuals were residing with parents who reported poor MH; 1.6% reported poor MH themselves, and 0.04% (*n* = 260) died by suicide. Living with a parent with poor MH increased the odds of offspring poor MH (OR = 2.80, 95% CI 2.59–3.03). After adjustment for age, gender, physical illness, socio-economic status and own MH, children living with 1 parent with poor MH were 76% more likely to die by suicide compared to children of parents who did not report poor MH (OR = 1.76, 95% CI 1.31–2.36). The effect size increased for children living with 2 parents with poor MH, and was higher in children aged under 24 years.

**Conclusions:**

Living with a parent with poor MH is a significant risk factor for offspring poor MH and suicide, even after adjustment for personal MH status. When treating mental ill-health in parents, potential interventions for their children should also be considered.

## Introduction

Suicide is a major public health concern with death by suicide increasing worldwide, especially amongst younger people (Gureje *et al*., 2011; World Health Organization, 2014). Approximately 800 000 people die by suicide annually, and it is the leading cause of death for males and females aged 20–34 years in the UK (World Health Organization, 2017; Office for National Statistics, 2020). However, our understanding of factors influencing suicidal behaviours is limited, and it is of vital importance to understand who is most at risk to better target interventions. Mental ill-health is one of the strongest predictors of suicide risk, with approximately 90% of those who attempt or complete suicide meeting diagnostic criteria for a mental disorder (Cavanagh *et al*., 2003; Bertolote *et al*., 2004; Windfuhr and Kapur, 2011). A recent systematic review concluded that the risk of suicide-related thoughts (SRTs) and suicide-related behaviours (SRBs), including suicide attempts, is significantly increased in those exposed to paternal SRTs and SRBs, or parental suicide (Goodday *et al*., 2019). Earlier studies of adopted children and twin studies posit that these associations are driven by genetic risk factor transmission (Qin *et al*., 2002; Sørensen *et al*., 2009; Gureje *et al*., 2011), but more recent evidence highlights the significant role of exposure to adverse childhood experiences including parental psychopathology (Wender *et al*., 1986; Glowinski *et al*., 2001; Roy and Segal, 2001). However, there is a paucity of research on the association between parental mental health (MH) and death by suicide among offspring.

The World Health Organization's MH surveys suggest that parental mental ill-health increases the risk of offspring suicide, though this was based not only on self-report of five ‘MH’ conditions including major depression, panic disorder and generalised anxiety disorder, but also substance dependence and antisocial behaviour (e.g. illegal behaviour, arrest and imprisonment) (Gureje *et al*., 2011). A Danish population cohort study observed a two-fold increase in suicide risk amongst offspring of parents with a history of psychiatric inpatient admissions, with a six-fold increase when both rather than one parent was affected (Webb *et al*., 2007). Two other Danish population-based case–control studies also identified parental psychiatric admission or suicide as substantial risk factors for suicide risk in offspring, and a Swedish case–control study showed that the offspring of parents with schizophrenia had a significantly increased risk of suicide, even after accounting for socio-economic status and offspring mental illness (Agerbo *et al*., 2002; Stenager and Qin, 2008; Ljung *et al*., 2013, 2013). However, although the current body of evidence suggests a significant association between parental psychopathology and offspring suicide risk, the derivation of parental MH status from psychiatric inpatient records limits the generalisability of these findings, potentially excluding individuals with less severe mental ill-health conditions and as such, the relationship between more common psychopathologies and offspring MH and suicide risk has yet to be elucidated (Mittendorfer-Rutz *et al*., 2008).

### Aims

The aims of this study were to identify the entire population of parents enumerated in the 2011 Census and examine their MH, and the MH and subsequent suicide risk amongst their offspring. During the study period, Northern Ireland had the highest age-standardised rate of suicide across the UK and Ireland, with 17.8 suicide deaths/100 000 compared to 11.8 and 12.1/100 000, respectively, making it an important setting for targeted suicide research and the advancement of suicide prevention strategies (ONS, 2013; Central Statistics Office, 2014; NISRA, 2017). This is the first population-wide cohort study analysing the risk of poor MH and suicide in offspring given parental MH status in the UK.

## Methods

This population-wide cohort study linked 2011 Census returns and mortality records to determine whether living with parents reporting poor MH is linked to offspring's self-reported poor MH, or increased risk of death by suicide.

### Data sources

The Northern Ireland Mortality Study (NIMS) links mortality records from the General Register's Office (GRO) to Census returns for the entire enumerated population. The Census is a mandatory questionnaire administered to all households in NI decennially and collects information on household characteristics and individual-level data on the demographics, health, education and employment of residents via self-report. NIMS and the linkage processes are described elsewhere (O'Reilly *et al*., 2012). For this study the March 2011 Census returns were linked with mortality records from the GRO until the end of 2016, allowing for 69 months of follow-up. The linked data were anonymised, held in a registered secure environment for analysis by the research team. The use of the NIMS for research was approved by the Office for Research Ethics Committees Northern Ireland (ORECNI).

### Identifying parents

The Census contains information on both individuals and on household structure enabling the identification of parent-child relationships. We limited the analysis to individuals living with a parent, regardless of age so households containing children and at least one parent were flagged and all other households dropped from the analysis. To clarify the association between MH of parents and children without the potential additional influence of grandparent MH, households in which children were identified as being parents themselves were excluded from the analysis. This resulted in the exclusion of 10 584 individuals.

### Cohort attributes

All personal characteristics of parents and children were drawn from the Census, as were other cohort attributes known to be associated with poor MH and suicide risk such as religion (Protestant, Roman Catholic, Other/none), physical illness (‘*Are your day to day activities limited because of a health problem or disability which has lasted, or is expected to last, at least 12 months*?’ Respondents could identify ‘Yes, limited a lot’, ‘Yes, limited a little’ or ‘No’), and socioeconomic status, which was assessed using a combination of housing tenure and the capital value of the property (Cohen *et al*., 1995). Tenure was Census-based (owner occupiers, private renting or social renting); Capital value had been derived by Land and Property Services which determined the level of local tax payable by each household. These data were linked and combined with tenure to produce an eight-fold classification defining tenure/taxable value of the property (private renting; social renting; and for owner-occupiers, five groups ranging from less than £75k to over £250k) (Table 1).

**Table 1.** Demographic characteristics of individuals who live at home with at least one parent and percentage of those who have ⩾1 parent with poor mental health (*n* =  618 970)

		Pop *N* = 618 970	Pop %	Poor parental mental health %	*p*-Value
Gender	Male Female	332 882 286 088	53.8 46.2	11.7 11.5	0.009
Age (years)	<16 17–23 24+	373 785 140 847 104 338	60.4 22.8 16.9	11.4 13.0 10.5	<0.001
Single parent household	No Yes	446 795 172 175	72.2 27.8	10.2 15.2	<0.001
Religion	Protestant Catholic Other/none	220 516 271 600 126 854	35.6 43.9 20.5	10.7 12.4 11.3	<0.001
Limiting long-term illness	No A little A lot	576 478 21 714 20 778	93.1 3.5 3.4	10.9 20.4 21.1	<0.001
House value & tenure	>250 k 200–249 k 150–199 k 100–149 k 75–99 k <75 k Private renting Social renting Non-response (missing/edited)	44 938 46 727 92 451 139 997 75 296 44 006 81 137 73 741 20 677	7.3 7.6 14.9 22.6 12.2 7.1 13.1 11.9 3.3	4.6 6.3 7.8 9.8 12.1 12.7 15.4 23.2 7.1	<0.001
Deprivation	Most affluent 2 3 4 Most deprived non-response (missing/edited)	117 807 116 205 118 409 120 193 117 032 29 324	19.0 18.8 19.1 19.4 18.9 4.7	7.6 9.5 10.8 13.3 17.5 8.4	<0.001
Personal poor mental health	No Yes	608 929 10 041	98.4 1.6	11.3 30.9	<0.001
Dead	No Yes	617 396 1574	99.7 0.3	11.6 12.1	0.536
Suicide	No Yes	618 710 260	99.96 0.04	11.6 23.9	<0.001

*χ*^2^ test of independence examining the relationship between offspring demographic characteristics and poor parental health.

### Area characteristics

Area-level deprivation was assigned using the ‘Income’ domain of the Northern Ireland Index of Multiple Deprivation (NIMDM) which is based on the proportion of residents on means-tested benefits (Northern Ireland Statistics and Research Agency, 2010). Areas were defined as 890 homogenous groups of approximately 2000 individuals and were ranked from least deprived to most deprived, then split into equal quintiles.

### MH status

The 2011 Census in Northern Ireland was unique in the UK in that it asked a detailed question on the presence of chronic health conditions. Individuals were asked ‘*Do you have any of the following conditions which have lasted, or are expected to last, at least 12 months*?’ and asked to tick all of a range of conditions that applied (Wright *et al*., 2017). Those who identified as having ‘*An emotional, psychological or MH condition (such as depression or schizophrenia)*’ were defined as having poor MH. This indicator has face validity based on its demographic distribution which mirrors known associations with MH, correlational validity as it is associated with receipt of psychotropic medication, and predictive validity as it is a strong predictor of death by suicide (Tseliou *et al*., 2015; Rosato *et al*., 2019; Onyeka *et al*., 2021).

### Mortality data

Information on the date and cause of deaths between April 2011 and December 2016 were based on official death records and linked probabilistically to the Census data as described in detail elsewhere (O'Reilly *et al*., 2008). In keeping with usual practice in the UK, suicide deaths included both deaths recorded as ‘suicide’ and deaths classified as ‘undetermined intent’ (ICD-10 code: X60-X84; Y10-Y34; Y87.0; Y87.2) (Linsley *et al*., 2001).

### Analysis strategy

The Census identified children residing with their parents and whether 0, 1 or 2 of their parents reported poor MH. Descriptive statistics recorded the socio-demographic characteristics and variations in health status and mortality of individuals stratified according to parental MH. Logistic regression compared the risks associated with both poor MH and suicide given parental MH status. Ordinary Logistic Regression assumes that the residuals are independent. The ‘cluster’ option in Stata 15.1 was used to account for the fact that the observations are clustered into households and may therefore be correlated within households, but independent between. This was enabled by the presence of a unique Census household identifier. Sensitivity analyses were carried out stratifying the children in younger (0–23 years) and older (⩾24 years) age groups to determine if the associations differed given the age of the child resident with their parent. This stratification differentiates between adults (⩾24 years) and children/young people (⩽23years). Whilst there is no universally accepted definition of ‘adolescence’ the United Nations defines adolescence as 15–24 years (United Nations, 2020). For this study it is assumed that by the age 24 years most young people should have completed education and be living independently.

## Results

### Characteristics of households with at least one parent with poor MH

The final cohort comprised 618 970 offspring living with their parents at the time of the 2011 Census. In total, 71 632 children (11.6%) were living with at least one parent who reported poor MH; 66 936 children (10.8%) were living with only one parent with poor MH, while 4696 (0.76%) were living with two parents with poor MH. The prevalence of parental poor MH was higher in single-parent families (15.2% vs 10.2%) (Table 1). Households affected by poor parental MH were characteristically more socially deprived, and were more than twice as common amongst families living in the more disadvantaged areas (17.5% vs 7.6%) and five times as common for families living in socially rented accommodation compared to those in larger owner-occupied homes (23.2% vs 4.6%).

### Offspring MH and relationship to parental MH

Although the majority of children lived in households not affected by poor parental MH, almost 1-in-8 children (11.6%) lived with one or more parents with poor MH. A total of 10 041 (1.6%) children reported poor MH and almost a third also lived with a parent with poor MH. Offspring poor MH was more prevalent in those living with a parent who reported poor MH compared to those who did not report poor MH (4.3% vs 1.3%). However, the majority of children living with a parent with poor mental did not report poor MH (95.7%).

In an unadjusted logistic regression model (Table 2), children living with one parent with poor MH were three-times more likely to report poor MH compared to children of parents not reporting poor MH (OR = 3.36, 95% CI 3.20–3.52). This increased to six-fold where both parents had poor MH (OR = 6.04, 95% CI 5.34–6.83). This association was marginally attenuated with further adjustment for sociodemographic factors and offspring physical illness. In the fully adjusted model, offspring living in households where one parent had poor MH had almost three-times the odds of also having poor MH compared to their peers whose parents who did not report poor MH (OR = 2.79, 95% CI 2.64–2.95), increasing to five-fold when both parents had poor MH (OR = 5.25, 95% CI 4.57–6.02).

**Table 2.** Logistic regression models determining the odds of poor mental health given parental mental health status

		Model 1	Model 2	Model 3	Model 4
Parental poor mental health	No 1 Poor mental health 2 + Poor mental health	1.00 3.36 (3.20–3.52) 6.04 (5.34–6.83)	1.00 3.91 (3.72–4.11) 9.24 (8.16–10.47)	1.00 2.97 (2.81–3.14) 5.84 (5.10–6.70)	1.00 2.79 (2.64–2.95) 5.25 (4.57–6.02)
Gender	Female Male		1.00 1.26 (1.21–1.32)	1.00 1.08 (1.03–1.13)	1.00 1.06 (1.01–1.11)
Age (continuous)	Age		1.08 (1.08–1.08)	1.05 (1.05–1.05)	1.05 (1.05–1.06)
Single parent household	No Yes		1.00 1.69 (1.61–1.78)	1.00 1.36 (1.30–1.44)	1.00 1.19 (1.13–1.26)
Religion	Protestant Catholic Other/none		1.00 1.08 (1.03–1.14) 1.08 (1.02–1.15)	1.00 1.04 (0.98–1.09) 1.22 (1.14–1.30)	1.00 1.01 (0.96–1.07) 1.26 (1.17–1.35)
Limiting long-term illness	No A little A lot			1.00 17.08 (16.04–18.17) 38.47 (36.37–40.69)	1.00 16.28 (15.29–17.33) 36.19 (34.18–38.31)
House value & tenure	>250 k 200–249 k 150–199 k 100–149 k 75–99 k <75 k Private renting Social renting Non-response (missing/edited)				1.00 0.97 (0.82–1.15) 1.10 (0.95–1.26) 1.16 (1.02–1.33) 1.26 (1.09–1.45) 1.21 (1.05–1.41) 1.40 (1.21–1.61) 1.62 (1.41–1.86) 0.90 (0.73–1.10)
Deprivation	Most affluent 2 3 4 Most deprived Non-response (missing/edited)				1.00 0.90 (0.82–0.98) 0.87 (0.80–0.95) 0.95 (0.88–1.04) 1.02 (0.93–1.11) 0.53 (0.44–0.65)

Figures represent odds ratios (95% confidence intervals).

Further examination showed that the association between parental and offspring MH was not related to offspring age (online Supplementary Table 1). The ORs of poor MH for offspring under 24 years were OR = 2.64 (95% CI 2.43–2.86) for those living with one parent with poor MH, and OR = 5.23 (95% CI 4.31–6.36) for those living with two parents with poor MH. For those aged 24 years or over, the ORs were OR = 2.53 (95% CI 2.33–2.74) and OR = 4.90 (95% CI 3.95–6.08), respectively.

Although a stratified analysis revealed slightly increased odds of offspring with poor MH where the mother rather than the father was the parent affected by poor MH (OR = 2.84, 95% CI 2.67–3.03 and OR = 2.63, 95% CI 2.38–2.92 respectively), this difference was not statistically significant (online Supplementary Table 2).

### Parental MH and offspring suicide risk

During follow-up, 1574 of our offspring cohort died, 260 (16.5%) by suicide. Of those who died by suicide, 18.5% had reported personal poor MH in the Census and 23.8% had been living in a household where at least one parent had poor MH. The fully adjusted logistic regression model (online Supplementary Table 3) showed that the biggest risk factors for suicide amongst offspring were male sex (OR_adj_ 4.70; 95% CI 3.28–6.73) and having poor personal MH (OR_adj_ 4.31; 95% CI 2.72–6.85). However, there was also a significant association between parental MH and offspring suicide risk. In the unadjusted model (Table 3), the odds of suicide where one parent had poor MH were twice that observed for those living in households who did not report parental poor MH (OR = 2.36, 95% CI 1.75–3.16), and this increased to OR = 2.95 (95% CI 1.21–7.16) where both parents had poor MH. These risks were moderately attenuated with further adjustment for sociodemographic characteristics and physical ill-health, to 1.94 (95% CI 1.45–2.60) and 2.59 (95% CI 1.06–6.32) respectively. The final model shows that the relationship between parental MH and offspring suicide persisted even after adjustment for offspring MH (one parent affected: OR = 1.76, 95% CI 1.31–2.36, two parents affected: OR = 2.18, 95% CI 0.89–5.34).

**Table 3.** Logistic regression models determining the odds of death by suicide given parental mental health status and sex of affected parent

		Model 1	Model 2	Model 3	Model 4	Model 5
Parental poor mental health	No 1 Poor mental health 2 + Poor mental health	1.00 2.36 (1.75–3.16) 2.95 (1.21–7.16)	1.00 2.33 (1.74–3.12) 3.54 (1.45–8.65)	1.00 2.15 (1.61–2.87) 3.09 (1.26–7.57)	1.00 1.94 (1.45–2.60) 2.59 (1.06–.32)	1.00 1.76 (1.31–2.36) 2.18 (0.89–5.34)
Parental poor mental health	No Mother poor mental health Father poor mental health Both poor mental health	1.00 2.30 (1.65–3.21) 2.53 (1.50–4.28) 2.95 (1.21–7.16)	1.00 2.25 (1.62–3.13) 2.56 (1.50–4.36) 3.55 (1.45–8.68)	1.00 2.06 (1.48–2.86) 2.37 (1.39–4.03) 3.09 (1.26– 7.57)	1.00 1.87 (1.35–2.60) 2.14 (1.25–3.67) 2.59 (1.06–6.33)	1.00 1.69 (1.22– 2.35) 1.95 (1.13–3.35) 2.20 (0.90–5.37)

Figures represent odds ratios (95% confidence intervals).

Model 1: unadjusted.

Model 2: + gender, age in years (continuous), single-parent household, and religion.

Model 3: + limiting long-term illness.

Model 4: + house value and tenure, and deprivation quintile.

Model 5: + personal mental health status.

The risk of suicide in offspring did not differ significantly according to which parent was affected, the risk being ORadj = 1.95 (95% CI 1.13–3.35) when the father had poor MH, compared to ORadj = 1.69 (95% CI 1.22–2.35) for those living with a mother with poor MH.

157 deaths by suicide were recorded among the 514 475 (0.03%) individuals aged under 24 years, whilst 103 suicides were reported among the 104 235 (0.1%) individuals aged 24 + years. Further stratification of logistic regression models revealed a differential impact of parental MH on suicide risk by age of offspring (Table 4). Amongst those aged under 24 years, the odds of suicide were increased 1.5-fold (ORadj = 1.54, 95% CI 1.06–2.25) where one parent had poor MH, increasing to ORadj = 2.80 (95% CI 1.02–7.65) where both parents reported poor MH. However, the observed association between poor parental MH and offspring suicide was much lower and not statistically significant in those aged 24+ years (one parent affected: OR = 1.37, 95% CI 0.83–2.26, two parents affected: OR = 0.73, 95% CI 0.10–5.24).

**Table 4.** Logistic regression models determining the odds ratios of death by suicide given parental mental health status, stratified by age

		≤23 years (*n* = 514 632)	≥24 years (*n* = 104 338)
Parental poor mental health	No 1 Poor mental health 2 + Poor mental health	1.00 1.54 (1.06–2.25) 2.80 (1.02–7.65)	1.00 1.37 (0.83–2.26) 0.73 (0.10- 5.24)
Gender	Female Male	1.00 4.12 (2.72–6.25)	1.00 6.33 (3.07–13.06)
Age in years (continuous)		1.20 (1.17–1.23)	1.00 (0.97–1.02)
Single parent household	No Yes	1.00 1.83 (1.28–2.63)	1.00 0.82 (0.53–1.26)
Religion	Protestant Catholic Other/none	1.00 1.40 (0.95– 2.06) 1.01 (0.62–1.63)	1.00 1.27 (0.82–1.99) 1.35 (0.77–2.37)
Limiting long-term illness	No Yes	1.00 0.96 (0.50–1.85)	1.00 2.52 (1.47–4.30)
House value & tenure	>250 k 200–249 k 150–199 k 100–149 k 75–99 k <75 k Private renting Social renting Non-response (missing/edited)	1.00 1.14 (0.35–3.74) 1.50 (0.55–4.12) 1.23 (0.45–3.34) 2.00 (0.73–5.50) 1.66 (0.56–4.93) 2.48 (0.90–6.83) 2.05 (0.74–5.64) 3.03 (0.95–9.67)	1.00 0.27 (0.03–2.56) 1.10 (0.30- 4.01) 1.58 (0.48–5.20) 1.01 (0.29- 3.54) 1.42 (0.41–4.91) 2.04 (0.60- 7.30) 1.36 (0.39–4.78) 0.46 (0.05–4.39)
Deprivation	Affluent Deprived	1.00 1.49 (1.00–2.22)	1.00 1.45 (0.97–2.16)
Personal poor mental health	No Yes	1.00 6.07 (3.03–12.18)	1.00 2.90 (1.64–5.14)

Figures represent odds ratios (95% confidence intervals).

## Discussion

This population-wide study demonstrates that living with a parent with poor MH is associated with a significantly increased risk of offspring with poor MH and death by suicide. Almost one-in-eight children in NI were living with at least one parent with poor MH and these individuals were twice as likely to die by suicide, even after accounting for physical health, household socio-economic status and personal MH status.

### Effect of parental MH on child MH

Although the aetiology of child psychopathology is multifactorial, the available literature indicates that exposure to parental psychopathology is a key determinant of mental disorders in children (McLaughlin *et al*., 2012; Rasic *et al*., 2014; Bellina *et al*., 2020). In the current study, there was a very strong relationship between the MH of parents and co-resident children, with children almost three-times more likely to report poor MH when one parent had poor MH, increasing to a 5-fold increase when both parents reported poor MH. This compares favourably with the existing body of literature; in a recent meta-analysis, Rasic *et al*., observed more than a two-fold increase in the risk of mental disorders in offspring of parents with severe mental illness, with even larger estimates observed for offspring with schizophrenia and bipolar disorder (Rasic *et al*., 2014). Direct comparison of the current study is however challenging given the methodological heterogeneity across studies, including disparities in the measures used to identify mental illness, the age of offspring examined and the MH condition examined. Many of the previous studies also lacked statistical power or information on the MH status of the second parent.

In the last decade, our understanding of potential mechanisms for the familial transmission of mental illness has advanced with the emergence of genetically informed studies. Systematic reviews provide substantial evidence of the genetic transmission of MH disorders, including schizophrenia and depression (McAdams *et al*., 2014; Jami *et al*., 2021), though it is also clear that psychopathology may be transmitted through the shared rearing environment and through unique parent-child interactions. Parenting practices and family functioning have been highlighted as the primary drivers of environmental transmission of psychopathology and these factors are strongly correlated with offspring psychological wellbeing, with healthy offspring development heavily dependent on parental health and wellbeing (Berg-Nielsen *et al*., 2002; Bradley and Vandell, 2007; Bellina *et al*., 2020). In this administrative data-based study we were unable to contribute any insight into whether the increased risk for poor MH is a direct result of a shared genetic susceptibility, or an indirect effect of the impact of parental poor MH on the child's upbringing, from exposure to common stressors, witnessing mental ill-health episodes or the subsequent parenting style or parental involvement.

### Effect of parental MH on child suicide

Personal poor MH remained the strongest independent risk factor for death by suicide in offspring even after adjustment for parental MH, contributing to more than a three-fold increase in the odds of suicide compared to those who did not report poor MH. This observation is consistent with findings from Danish studies where an up to 39-fold increase in the risk of suicide in offspring with a personal psychiatric hospitalisation was observed (Stenager and Qin, 2008; Sørensen *et al*., 2009). Although a smaller magnitude of the effect was observed in this study, it is highly plausible that the identification of poor MH from a community-based self-reported measure would capture individuals with a wider range of mental ill-health conditions, including those considered less severe when compared to those requiring psychiatric inpatient admission, as is the case in the majority of studies examining this association. It is universally recognised by the authors of these studies that most cases with mild to moderate mental disorders are diagnosed and treated by general practitioners and will not be observed in such centralised psychiatric registers (Mors *et al*., 2011).

The aetiology of the increased risk of mental illness and increased suicide risk in offspring of parents with mental illness are closely interlinked. Support for the implication of genetic transmission of risk for suicidal behaviours has been provided using a variety of study methodologies, including family studies, twin studies and adoptive studies (Mirkovic *et al*., 2016). Examination of the available evidence indicates significant heritability of suicide, estimated at an aggregated 45%, but this is highly dependent on the presence of psychiatric illness (Mirkovic *et al*., 2016). In addition, a wide range of environmental factors has been indicated in the development of suicidal behaviours, with the majority of studies attributing the increased risk to a wide range of early adverse experiences including maltreatment, interpersonal loss (e.g. parental death, separation) socio-economic adversity and family dysfunction (e.g. parental mental illness, domestic violence, incarceration) (Sahle *et al*., 2021). Alterations in family functioning and parenting capacity owing to psychiatric illness may in turn increase exposure to these adverse childhood experiences, conferring a greater risk of internalising and externalising behaviours in exposed offspring(Hughes *et al*., 2017; Petruccelli *et al*., 2019), which are important risk factors for SRTs and behaviours among young people (Goodday *et al*., 2019).

We observed an elevated risk of suicide where both parents reported poor MH, which corroborates with Webb *et al*., who noted a 6-fold increase in offspring suicide where two parents had a recorded psychiatric inpatient record, compared to a 2–3-fold increase in those with one affected parent (Webb *et al*., 2007). While this may represent increased genetic potential it is also plausible that exposure to two parents with poor MH would also result in greater impairment of parenting capacity and a more dysfunctional family home environment. In order to disentangle this relationship, studies which account for possible genetic influences, such as adoption and children-of-twins designs are necessary. However, suicide is both a rare and sensitive outcome, thus achieving sufficient power to provide robust risk estimates is challenging, even in large population-wide studies.

Whilst the association between parental and offspring MH persisted for both younger and older offspring, the relationship between parental MH and offspring suicide remained significant only for those aged under 24 years. Few studies have examined the impact of parental psychopathology across sensitive life periods in children and adolescents, but this finding, together with those of Niederkotenthaler *et al*. (Niederkrotenthaler *et al*., 2012) support the hypothesis that early exposure to parental psychopathology during neuro-developmentally sensitive periods may increase risk of suicide. It is also plausible that the impact of environmental influences, including parental psychopathology, on offspring MH and suicidality may decrease with increasing age and independence from the family unit. It is also important to note that these older individuals may have moved out of the family home during follow-up, therefore lessening the observed parental-offspring associations. However, given that the confidence intervals were wide and cross unity, this observation may be the result of insufficient statistical power to detect the true association between the number of parents who reported poor MH and suicide risk among offspring aged 24+ years.

Studies suggest that the risk of suicide varies according to the parent affected. A recent systematic review concluded that the risk of SRBs and death by suicide in children was higher when it was the mother rather than the father who had poor MH though, for death by suicide alone the evidence for a differential impact of parental sex was less conclusive (Goodday *et al*., 2019). In two case–control studies using national registers in Denmark, maternal psychiatric hospitalisation was associated with a minimally increased risk of offspring suicide compared to those with a record of paternal psychiatric hospitalisation(Webb *et al*., 2007; Stenager and Qin, 2008), and in a Swedish population-based case–control study, maternal schizophrenia was associated with slightly higher odds of offspring suicide than paternal schizophrenia (Ljung *et al*., 2013). However, it is unclear whether the differential risk estimates observed in these studies were statistically significantly different. The current study does not resolve the issue. The elevated risk of offspring with poor MH was marginally greater for individuals living with a mother with poor MH, but conversely, the risk of offspring suicide was greater to in those living with a father with poor MH. Previous research on parenting and child psychopathology suggests that although the quality of paternal-child relationships strongly predicts the emotional and behavioural outcomes of offspring, the impact of maternal influence supersedes that of the father, presumably as the primary burden of parental care is adopted by the mother (Flouri, 2010).

### Strengths and limitations

This population-wide cohort study provided a unique opportunity to examine the relationship between parental MH and offspring psychopathology and suicide risk in a typically hard to reach population. The self-reported indicator of poor MH encompassed a wider range of psychopathology than studies based on admission to a psychiatric hospital, making these findings more generalisable to the wider body of individuals with mental ill-health. Although admission data has the benefit of high specificity, it excludes the much larger proportion of psychiatric ill-health that is managed in the community. Additionally, population surveys which use validated diagnostic instruments, though arguably the most accurate measurement of MH, are resource intensive, require a clinical application or specialist interviewer training and cannot cover more than a small proportion of the population. However, as the Census measure used included only those with conditions that had or were expected to last at least twelve months, people with less protracted episodes of poor MH will not have been included. Furthermore, a self-report measure, while democratising the identification process, is subject to reporting bias especially related to the continuing stigma surrounding mental illness. However, this indicator has face validity based on its demographic distribution which mirrors known associations with MH, correlational validity as it is associated with receipt of psychotropic medication, and predictive validity as it is a strong predictor of death by suicide (Tseliou *et al*., 2015; Rosato *et al*., 2019; Onyeka *et al*., 2021).

As parental and offspring MH were measured at the same time point, establishing causal order by accounting for reverse causality was not possible. There is likely some degree of bidirectionality to this association, however, the association persisted with adjustment for person MH status, and only 1.62% of children reported poor MH despite 11.6% living with an affected parent, providing some indication of the direction of the association. This also limited the statistical modelling to logistic regression, however, there is an argument that when the outcome is rare and the follow-up period is short, the regression coefficients obtained through logistic regression approximate those obtained through Cox regression (Green and Symons, 1983; Ingram and Kleinman, 1989; Ingram *et al*., 1990). Finally, while Census-based studies provide population coverage, they lack depth and additional information on important household factors, such as substance dependencies in parents or exposure to adverse childhood experiences which would have contributed to our understanding of factors that may mediate these associations. Owing to the study design, it was not possible to examine the extent to which the observed parent-offspring associations could be explained by shared genetic factors. As such, further investigation into the mechanisms underpinning these associations is warranted.

## Conclusion

Parental poor MH is strongly associated with offspring suicide risk even after adjustment for personal MH status, especially in children aged under 24 years. However, personal MH remains the greatest predictor of suicide risk. Given the high prevalence of parental psychopathology in households in Northern Ireland, early identification of children and families at excess risk is crucial in the treatment of mental ill-health and the prevention of suicide. These findings indicate that the wider household effects of poor MH should be taken into consideration when treating parents with mental illness and treatment plans should consider potential interventions for their children also, particularly in families with children under 24 years.

## Data Availability

The de-identified research dataset was made available only to the approved research team within the secure setting at the Northern Ireland Statistics and Research Agency (NISRA).

## Supplementary material

For supplementary material accompanying this paper visit https://doi.org/10.1017/S2045796022000063. click here to view supplementary material

## References

[ref1] Agerbo E, Nordentoft M and Mortensen PB (2002) Familial, psychiatric, and socioeconomic risk factors for suicide in young people: nested case–control study. British Medical Journal 325, 74–77. 12114236 10.1136/bmj.325.7355.74 PMC117126

[ref2] Bellina M, Grazioli S, Garzitto M, Mauri M, Rosi E, Molteni M, Brambilla P and Nobile M (2020) Relationship between parenting measures and parents and child psychopathological symptoms: a cross-sectional study. BMC Psychiatry 20, 377. 32680486 10.1186/s12888-020-02778-8 PMC7367317

[ref3] Berg-Nielsen TS, Vikan A and Dahl AA (2002) Parenting related to child and parental psychopathology: a descriptive review of the literature. Clinical Child Psychology and Psychiatry 7, 529–552.

[ref4] Bertolote JM, Fleischmann A, De Leo D and Wasserman D (2004) Psychiatric diagnoses and suicide: revisiting the evidence. Crisis 25, 147–155. 15580849 10.1027/0227-5910.25.4.147

[ref5] Bradley RH and Vandell DL (2007) Child care and the well-being of children. Archives of Pediatrics and Adolescent Medicine 161, 669–676. 17606830 10.1001/archpedi.161.7.669

[ref6] Cavanagh JTO, Carson AJ, Sharpe M and Lawrie SM (2003) Psychological autopsy studies of suicide: a systematic review. Psychological Medicine 33, 395–405. 12701661 10.1017/s0033291702006943

[ref7] Central Statistics Office (2014) Suicide Statistics 2011. Available at https://www.cso.ie/en/releasesandpublications/er/ss/suicidestatistics2011/. (Accessed 3 March 2021).

[ref8] Cohen G, Forbes J and Garraway M (1995) Interpreting self-reported limiting long term illness. BMJ (Clinical Research Edition) 311, 722–724. 10.1136/bmj.311.7007.722 PMC2550719 7549686

[ref9] Flouri E (2010) Fathers’ behaviors and children's psychopathology. Clinical Psychology Review 30, 363–369. 20149945 10.1016/j.cpr.2010.01.004

[ref10] Glowinski AL, Bucholz KK, Nelson EC, Fu Q, Madden PAF, Reich W and Heath AC (2001) Suicide attempts in an adolescent female twin sample. Journal of the American Academy of Child and Adolescent Psychiatry 40, 1300–1307. 11699804 10.1097/00004583-200111000-00010 PMC1474069

[ref11] Goodday SM, Shuldiner J, Bondy S and Rhodes AE (2019) Exposure to parental psychopathology and offspring's risk of suicide-related thoughts and behaviours: a systematic review. Epidemiology and Psychiatric Sciences 28, 179–190. 28748774 10.1017/S2045796017000397 PMC6998933

[ref12] Green MS and Symons MJ (1983) A comparison of the logistic risk function and the proportional hazards model in prospective epidemiologic studies. Journal of Chronic Diseases 36, 715–723. 6630407 10.1016/0021-9681(83)90165-0

[ref13] Gureje O, Oladeji B, Hwang I, Chiu WT, Kessler RC, Sampson NA, Alonso J, Andrade LH, Beautrais A, Borges G, Bromet E, Bruffaerts R, de Girolamo G, de Graaf R, Gal G, He Y, Hu C, Iwata N, Karam EG, Kovess-Masféty V, Matschinger H, Moldovan MV, Posada-Villa J, Sagar R, Scocco P, Seedat S, Tomov T and Nock MK (2011) Parental psychopathology and the risk of suicidal behavior in their offspring: results from the world mental health surveys. Molecular Psychiatry 16, 1221–1233. 21079606 10.1038/mp.2010.111 PMC3142278

[ref14] Hughes K, Bellis MA, Hardcastle KA, Sethi D, Butchart A, Mikton C, Jones L and Dunne MP (2017) The effect of multiple adverse childhood experiences on health: a systematic review and meta-analysis. The Lancet Public Health 2, e356–e366. 29253477 10.1016/S2468-2667(17)30118-4

[ref15] Ingram DD and Kleinman JC (1989) Empirical comparisons of proportional hazards and logistic regression models. Statistics in Medicine 8, 525–538. 2727473 10.1002/sim.4780080502

[ref16] Ingram DD, Kleinman JC and Mock P (1990) Empirical comparisons of proportional hazards and logistic regression models. Statistics in Medicine 9, 463–464. 2362982 10.1002/sim.4780090417

[ref17] Jami ES, Hammerschlag AR, Bartels M and Middeldorp CM (2021) Parental characteristics and offspring mental health and related outcomes: a systematic review of genetically informative literature. Translational Psychiatry 11, 197. 33795643 10.1038/s41398-021-01300-2 PMC8016911

[ref18] Linsley KR, Schapira K and Kelly TP (2001) Open verdict v. suicide – importance to research. The British Journal of Psychiatry 178, 465–468. 11331564 10.1192/bjp.178.5.465

[ref19] Ljung T, Lichtenstein P, Sandin S, D'Onofrio B, Runeson B, Långström N and Larsson H (2013) Parental schizophrenia and increased offspring suicide risk: exploring the causal hypothesis using cousin comparisons. Psychological Medicine 43, 581–590. 22703756 10.1017/S0033291712001365 PMC3669221

[ref20] McAdams TA, Neiderhiser JM, Rijsdijk F V., Narusyte J, Lichtenstein P and Eley TC (2014) Accounting for genetic and environmental confounds in associations between parent and child characteristics: a systematic review of children-of-twins studies. Psychological Bulletin 140, 1138–1173. 24749497 10.1037/a0036416

[ref21] McLaughlin KA, Gadermann AM, Hwang I, Sampson NA, Al-Hamzawi A, Andrade LH, Angermeyer MC, Benjet C, Bromet EJ, Bruffaerts R, Caldas-de-Almeida JM, de Girolamo G, de Graaf R, Florescu S, Gureje O, Haro JM, Hinkov HR, Horiguchi I, Hu C, Karam AN and Kessler RC (2012) Parent psychopathology and offspring mental disorders: results from the WHO world mental health surveys. British Journal of Psychiatry 200, 290–299. 10.1192/bjp.bp.111.101253 PMC3317036 22403085

[ref22] Mirkovic B, Laurent C, Podlipski MA, Frebourg T, Cohen D and Gerardin P (2016) Genetic association studies of suicidal behavior: a review of the past 10 years, progress, limitations, and future directions. Frontiers in Psychiatry 7, 1. 27721799 10.3389/fpsyt.2016.00158 PMC5034008

[ref23] Mittendorfer-Rutz F, Rasmussen F and Wasserman D (2008) Familial clustering of suicidal behaviour and psychopathology in young suicide attempters: a register-based nested case–control study. Social Psychiatry and Psychiatric Epidemiology 43, 28–36. 17934681 10.1007/s00127-007-0266-0

[ref24] Mors O, Perto GP, Preben and Mortensen BO (2011) The Danish psychiatric central research register. Scandinavian Journal of Public Health 39, 54–57. 21775352 10.1177/1403494810395825

[ref25] Niederkrotenthaler T, Floderus B, Alexanderson K, Rasmussen F and Mittendorfer-Rutz E (2012) Exposure to parental mortality and markers of morbidity, and the risks of attempted and completed suicide in offspring: an analysis of sensitive life periods. Journal of Epidemiology and Community Health 66, 233–239. 20924054 10.1136/jech.2010.109595

[ref26] Northern Ireland Statistics and Research Agency (2010) Northern Ireland Multiple Deprivation Measure 2010. Available at https://www.nisra.gov.uk/sites/nisra.gov.uk/files/publications/NIMDM_2010_Report_0.pdf (Accessed 3 March 2020).

[ref27] Northern Ireland Statistics and Research Agency (2017) Suicide Deaths. Available at https://www.nisra.gov.uk/publications/suicide-statistics (Accessed 3 March 2020).

[ref28] Office for National Statistics (2013) Suicides in the UK: 2011 Registrations. Available at https://www.ons.gov.uk/peoplepopulationandcommunity/birthsdeathsandmarriages/deaths/bulletins/suicidesintheunitedkingdom/2013-01-22 (Accessed 3 March 2020).

[ref29] Office for National Statistics (2020) Leading causes of death, UK: 2001 to 2018. Available at https://www.ons.gov.uk/peoplepopulationandcommunity/healthandsocialcare/causesofdeath/articles/leadingcausesofdeathuk/2001to2018. (Accessed 22 May 2021).

[ref30] Onyeka IN, O'Reilly D and Maguire A (2021) The association between self-reported mental health, medication record and suicide risk: a population-wide study. SSM – Population Health 13, 100749. 33665331 10.1016/j.ssmph.2021.100749 PMC7901032

[ref31] O'Reilly D, Rosato M and Connolly S (2008) Unlinked vital events in census-based longitudinal studies can bias subsequent analysis. Journal of Clinical Epidemiology 61, 380–385. 18313563 10.1016/j.jclinepi.2007.05.012

[ref32] O'Reilly D, Rosato M, Catney G, Johnston F and Brolly M (2012) Cohort description: the Northern Ireland Longitudinal Study (NILS). International Journal of Epidemiology 41, 634–641. 21296852 10.1093/ije/dyq271

[ref33] Petruccelli K, Davis J and Berman T (2019) Adverse childhood experiences and associated health outcomes: a systematic review and meta-analysis. Child Abuse and Neglect 97, 104127. 31454589 10.1016/j.chiabu.2019.104127

[ref34] Qin P, Agerbo E and Mortensen PB (2002) Suicide risk in relation to family history of completed suicide and psychiatric disorders: a nested case–control study based on longitudinal registers. Lancet (London, England) 360, 1126–1130. 10.1016/S0140-6736(02)11197-4 12387960

[ref35] Rasic D, Hajek T, Alda M and Uher R (2014) Risk of mental illness in offspring of parents With schizophrenia, bipolar disorder, and Major depressive disorder: a meta-analysis of family high-risk studies. Schizophrenia Bulletin 40, 28–38. 23960245 10.1093/schbul/sbt114 PMC3885302

[ref36] Rosato M, Tseliou F and O'Reilly D (2019) Unmet need for chronic mental ill health: a population-based record linkage study. International Journal of Population Data Science 4, 1122. 34095538 10.23889/ijpds.v4i1.1122 PMC8142951

[ref37] Roy A and Segal N (2001) Suicidal behavior in twins: a replication. Journal of Affective Disorders 66, 71–74. 11532534 10.1016/s0165-0327(00)00275-5

[ref38] Sahle BW, Reavley NJ, Li W, Morgan AJ, Yap MBH, Reupert A and Jorm AF (2021) The association between adverse childhood experiences and common mental disorders and suicidality: an umbrella review of systematic reviews and meta-analyses. European Child and Adolescent Psychiatry 1, 3. 10.1007/s00787-021-01745-2 33638709

[ref39] Sørensen HJ, Mortensen EL, Wang AG, Juel K, Silverton L and Mednick SA (2009) Suicide and mental illness in parents and risk of suicide in offspring. Social Psychiatry and Psychiatric Epidemiology 44, 748–751. 19169611 10.1007/s00127-009-0495-5

[ref40] Stenager K and Qin P (2008) Individual and parental psychiatric history and risk for suicide among adolescents and young adults in Denmark. Social Psychiatry and Psychiatric Epidemiology 43, 920–926. 18574540 10.1007/s00127-008-0385-2

[ref41] Tseliou F, Maguire A, Donnelly M and O'Reilly D (2015) The impact of childhood residential mobility on mental health outcomes in adolescence and early adulthood: a record linkage study. Journal of Epidemiology & Community Health 70, 278–285. 26475920 10.1136/jech-2015-206123

[ref42] United Nations (2020) World Youth Report. Available at https://www.un.org/development/desa/youth/wp-content/uploads/sites/21/2020/07/2020-World-Youth-Report-FULL-FINAL.pdf (Accessed 20 January 2021).

[ref43] Webb RT, Pickles AR, Appleby L, Mortensen PB and Abel KM (2007) Death by unnatural causes during childhood and early adulthood in offspring of psychiatric inpatients. Archives of General Psychiatry 64, 345–352. 17339523 10.1001/archpsyc.64.3.345

[ref44] Wender PH, Kety SS, Rosenthal D, Schulsinger F, Ortmann J and Lunde I (1986) Psychiatric disorders in the biological and adoptive families of adopted individuals with affective disorders. Archives of General Psychiatry 43, 923–929. 3753159 10.1001/archpsyc.1986.01800100013003

[ref45] Windfuhr K and Kapur N (2011) Suicide and mental illness: a clinical review of 15 years findings from the UK national confidential inquiry into suicide. British Medical Bulletin 100, 101–121. 21948337 10.1093/bmb/ldr042

[ref46] World Health Organization (2014) Preventing Suicide. A Global Imperative. Available at https://apps.who.int/iris/handle/10665/131056 (Accessed 3 March 2020).

[ref47] World Health Organization (2017) World Health Statistics 2017: Monitoring Health for The SDGs. Available at 10.1017/CBO9781107415324.004 (Accessed 3 March 2020).

[ref48] Wright DM, Rosato M and O'Reilly D (2017) Which long-term illnesses do patients find most limiting? A census-based cross-sectional study of 340,000 people. International Journal of Public Health 62, 939–947. 27942744 10.1007/s00038-016-0929-2 PMC5641274

