# Supplementary material for: Parental mental health and risk of poor mental health and death by suicide in offspring: a population-wide data-linkage study
Source: Epidemiol Psychiatr Sci. 2022 Apr 19;31:e25. doi: 10.1017/S2045796022000063 (PMC9069591; doi:10.1017/S2045796022000063)
Supplement: Supplementary file 1 [file S2045796022000063sup001.docx]

**Supplementary Tables**

**Table 1. Logistic Regression models determining the odds ratios of offspring poor mental health given parental mental health status, stratified by age. Figures represent odds ratios (95% confidence intervals)**

|  |  | **<=23 years**  **(n=514,632)** | **>=24 years**  **(n=104,338)** |
| --- | --- | --- | --- |
| **Parental Poor Mental Health** | **No**  **1 Poor mental health**  **2+ Poor mental health** | 1.00  2.64 (2.43- 2.86)  5.23 (4.31- 6.36) | 1.00  2.53 (2.33- 2.74)  4.90 (3.95- 6.08) |
| **Gender** | **Female**  **Male** | 1.00  0.97 (0.91- 1.04) | 1.00  1.05 (0.98- 1.12) |
| **Age in years** *(continuous)* |  | 1.16 (1.15- 1.17) | 1.01 (1.01- 1.01) |
| **Single Parent Household** | **No**  **Yes** | 1.00  1.51 (1.38- 1.64) | 1.00  1.36 (1.27- 1.46) |
| **Religion** | **Protestant**  **Catholic**  **Other/none** | 1.00  0.91 (0.84- 0.99)  1.25 (1.13- 1.38) | 1.00  1.01 (0.94- 1.08)  1.18 (1.08- 1.29) |
| **Limiting Long-term Illness** | **No**  **A little**  **A lot** | 1.00  20.27 (18.49- 22.22)  61.12 (56.27- 66.39) | 1.00  14.39 (13.23- 15.65)  25.32 (23.51- 27.27) |
| **House Value & Tenure** | **>250k**  **200-249k**  **150-199k**  **100-149k**  **75-99k**  **<75k**  **Private Renting**  **Social Renting**  **Non-response (missing/edited)** | 1.00  0.95 (0.75- 1.22)  1.10 (0.89- 1.35)  1.29 (1.06- 1.57)  1.31 (1.06- 1.62)  1.28 (1.02- 1.60)  1.64 (1.34- 2.02)  1.60 (1.30- 1.96)  1.00 (0.73- 1.37) | 1.00  0.99 (0.79- 1.24)  1.02 (0.85- 1.24)  1.04 (0.87- 1.25)  1.18 (0.98- 1.42)  1.15 (0.94- 1.39)  1.46 (1.19- 1.78)  1.61 (1.34- 1.95)  0.85 (0.65- 1.11) |
| **Deprivation** | **Most Affluent**  **2**  **3**  **4**  **Most Deprived**  **Non-response (missing/edited)** | 1.00  0.83 (0.73- 0.95)  0.82 (0.72- 0.92)  0.86 (0.76- 0.97)  0.92 (0.81- 1.04)  0.29 (0.22- 0.39) | 1.00  0.94 (0.84- 1.06)  0.90 (0.81- 1.01)  1.00 (0.90- 1.12)  1.07 (0.95- 1.20)  0.64 (0.50- 0.83) |

**Table 2: Logistic Regression models determining the odds of poor mental health given parental mental health status and parental sex. Figures represent odds ratios (95% confidence intervals)**

|  |  | **Model 1** | **Model 2** | **Model 3** | **Model 4** |
| --- | --- | --- | --- | --- | --- |
| **Parental Poor Mental Health** | **No**  **Mother poor mental health**  **Father poor mental health**  **Both poor mental health** | 1.00  3.47 (3.30- 3.66)  3.00 (2.74- 3.27)  6.04 (5.34- 6.83) | 1.00  4.04 (3.82- 4.27)  3.52 (3.21- 3.86)  9.20 (8.12- 10.43) | 1.00  3.02 (2.84- 3.21)  2.80 (2.53- 3.11)  5.83 (5.09- 6.69) | 1.00  2.84 (2.67- 3.03)  2.63 (2.38- 2.92)  5.24 (4.56- 6.01) |
| **Gender** | **Female**  **Male** |  | 1.00  1.26 (1.21- 1.32) | 1.00  1.08 (1.03- 1.13) | 1.00  1.06 (1.01- 1.11) |
| **Age** *(continuous)* | **Age** |  | 1.08 (1.08- 1.08) | 1.05 (1.05- 1.05) | 1.05 (1.05- 1.06) |
| **Single Parent Household** | **No**  **Yes** |  | 1.00  1.67 (1.59- 1.76) | 1.00  1.36 (1.29- 1.43) | 1.00  1.18 (1.12- 1.25) |
| **Religion** | **Protestant**  **Catholic**  **Other/none** |  | 1.00  1.08 (1.03- 1.14)  1.08 (1.02- 1.15) | 1.00  1.04 (0.98- 1.09)  1.22 (1.14- 1.31) | 1.00  1.01 (0.96- 1.07)  1.26 (1.18- 1.35) |
| **Limiting Long-term Illness** | **No**  **A little**  **A lot** |  |  | 1.00  17.07 (16.04- 18.17)  38.46 (36.36- 40.68) | 1.00  16.27 (15.28- 17.33)  36.18 (34.17- 38.30) |
| **House Value & Tenure** | **>250k**  **200-249k**  **150-199k**  **100-149k**  **75-99k**  **<75k**  **Private Renting**  **Social Renting**  **Non-response (missing/edited)** |  |  |  | 1.00  0.97 (0.82- 1.15)  1.10 (0.95- 1.26)  1.16 (1.02- 1.33)  1.26 (1.09- 1.45)  1.22 (1.05- 1.41)  1.40 (1.22- 1.62)  1.62 (1.41- 1.86)  0.90 (0.73- 1.10) |
| **Deprivation** | **Most Affluent**  **2**  **3**  **4**  **Most Deprived**  **Non-response (missing/edited)** |  |  |  | 1.00  0.90 (0.82- 0.98)  0.87 (0.80- 0.95)  0.95 (0.88- 1.04)  1.02 (0.93- 1.11)  0.53 (0.44- 0.65) |

**Table 3: Full Logistic Regression models determining the odds of death by suicide given parental mental health status. Figures represent odds ratios (95% confidence intervals)**

|  |  | **Model 1** | **Model 2** | **Model 3** | **Model 4** | **Model 5** |
| --- | --- | --- | --- | --- | --- | --- |
| **Parental Poor Mental Health** | **No**  **1 poor mental health**  **2+ poor mental health** | 1.00  2.36 (1.75-3.16)  2.95 (1.21-7.16) | 1.00  2.33 (1.74-3.12)  3.54 (1.45-8.65) | 1.00  2.15 (1.61-2.87)  3.09 (1.26-7.57) | 1.00  1.94 (1.45-2.60)  2.59 (1.06-6.32) | 1.00  1.76 (1.31-2.36)  2.18 (0.89-5.34) |
| **Gender** | **Female**  **Male** |  | 1.00  4.92 (3.44- 7.02) | 1.00  4.80 (3.36- 6.87) | 1.00  4.72 (3.30- 6.76) | 1.00  4.70 (3.28- 6.73) |
|  | **Age** |  | 1.05 (1.04- 1.06) | 1.04 (1.04- 1.05) | 1.04 (1.04- 1.05) | 1.04 (1.03- 1.05) |
| **Single Parent Household** | **No**  **Yes** |  | 1.00  1.53 (1.17- 2.01) | 1.00  1.45 (1.10- 1.91) | 1.00  1.17 (0.88- 1.55) | 1.00  1.15 (0.87- 1.54) |
| **Religion** | **Protestant**  **Catholic**  **Other/none** |  | 1.00  1.59 (1.19- 2.11)  1.29 (0.89- 1.85) | 1.00  1.58 (1.18- 2.10)  1.31 (0.91- 1.88) | 1.00  1.43 (1.07- 1.91)  1.28 (0.89- 1.85) | 1.00  1.41 (1.05- 1.88)  1.26 (0.87- 1.82) |
| **Limiting Long-term Illness** | **No**  **Yes** |  |  | 1.00  2.38 (1.77- 3.19) | 1.00  2.22 (1.65- 3.00) | 1.00  1.32 (0.88- 1.98) |
| **House Value & Tenure** | **>250k**  **200-249k**  **150-199k**  **100-149k**  **75-99k**  **<75k**  **Private Renting**  **Social Renting**  **Non-response (missing/edited)** |  |  |  | 1.00  0.78 (0.28- 2.15)  1.36 (0.61- 3.01)  1.44 (0.68- 3.08)  1.48 (0.67- 3.24)  1.55 (0.69- 3.50)  2.05 (0.93- 4.49)  1.88 (0.86- 4.11)  1.70 (0.63- 4.55) | 1.00  0.78 (0.28- 2.15)  1.37 (0.62- 3.03)  1.46 (0.69- 3.11)  1.48 (0.67- 3.25)  1.57 (0.70- 3.54)  2.02 (0.92- 4.42)  1.80 (0.82- 3.93)  1.73 (0.65- 4.62) |
| **Deprivation** | **Affluent**  **Deprived** |  |  |  | 1.00  1.48 (1.12- 1.96) | 1.00  1.46 (1.10- 1.94) |
| **Personal Poor Mental Health** | **No**  **Yes** |  |  |  |  | 1.00  4.31 (2.72- 6.85) |

Demographic characteristics of those individuals who live with at least one parent and die by suicide between April 2011 and December 2016.

|  |  | **Deaths by Suicide**  **N=260** | **Deaths by Suicide**  **%** | **p** |
| --- | --- | --- | --- | --- |
| **Parental Poor Mental Health** | **No**  **Yes** | 198  62 | 76.2  23.8 | <0.01 |
| **Gender** | **Female**  **Male** | 35  225 | 13.5  86.5 | <0.01 |
| **Age Group (years)** | **<16**  **17-23**  **24+** | 54  103  103 | 20.8  39.6  39.6 | <0.01 |
| **Single Parent Household** | **No**  **Yes** | 143  117 | 55.0  45.0 | <0.01 |
| **Religion** | **Protestant**  **Catholic**  **Other** | 72  137  51 | 27.7  52.7  20.5 | 0.01 |
| **Limiting Long-term Illness** | **No**  **Yes** | 194  66 | 74.6  25.4 | <0.01 |
| **Deprivation** | **Deprived**  **Not deprived/** **Non-response (missing/edited)** | 148  112 | 56.9  43.1 | <0.01 |
| **Personal Poor Mental Health** | **No**  **Yes** | 212  48 | 81.5  18.5 | <0.01 |

Chi-Square test of independence examining the relationship between demographic characteristics and death by suicide.
